# Odontogenic Keratocysts: Presentation and Surgical Outcome in a Sample of Sudanese Patients

**DOI:** 10.1155/2023/8763948

**Published:** 2023-10-12

**Authors:** Alaa Ayman Mohamed, Abdualhameed Abbas Babiker, Mazin Salah Khalfallah, Yousif Idris Eltohami

**Affiliations:** Department of Oral and Maxillofacial Surgery, Faculty of Dentistry, University of Khartoum, Khartoum, Sudan

## Abstract

**Background:**

Odontogenic keratocyst (OKC) is a benign intraosseous lesion relatively frequent in the oral cavity. It has a locally aggressive behavior and exhibits a high propensity to recur after treatment. The present study aimed to investigate the clinicoradiographic presentations and outcomes of surgical treatment of OKC at Khartoum Teaching Dental Hospital. *Material and Methods*. Fifty-five cases of OKC files at the Department of Oral and Maxillofacial Surgery in Khartoum Teaching Hospital between 2012 and 2022 were reviewed and studied using a descriptive prospective cross-sectional study. Data were analyzed using Statistical Package for the Social Sciences (SPSS) version 27.

**Results:**

Out of 55 cases studied, the mean age at the time of diagnosis was 30 ± 17.6 years; of them, 28 (50.9%) were males and 27 (49.1%) were females. The most common clinical manifestation was swelling (34.9%), followed by pain (28.0%). Sixty-five percent of the OKCs were located in the posterior mandible, and multilocular radiolucency (73.6%) was the most prevalent radiological finding. Enucleation with Carnoy's solution (CS) (55.2%) was the most common surgical modality that was used in more than half the patients, and only six patients had a recurrence. Marsupialization and segmental resection had no recurrence, while marginal resection and curettage had the highest recurrence rate of 20%. Patients with consanguineous parents had a higher recurrence rate (15%) compared with patients who had nonconsanguineous parents (8.6%). None of the patients died or had a malignant transformation.

**Conclusion:**

The most common location for the cyst was the mandible, and multilocular radiolucency was the most prevalent radiological finding. Enucleation with CS was the most commonly used surgical modality, used in more than half of the patients, with only six patients experiencing recurrence.

## 1. Introduction

Odontogenic keratocysts (OKCs) are cystic lesions appearing in the jaws, classified as developmental cysts arising from the dental lamina. They represent approximately 11% of all odontogenic cysts, and the age of onset has two peaks of maximum frequency, one between the second and third decades of life and the other, less intense, around the fifth decade. These cysts are more frequent in men, with a ratio of 1 : 1.4, and they are more common in the mandible, with a predilection for the angle and ramus. This lesion may occur in association with an impacted third molar. Compared to other odontogenic cysts, OKC has an infiltrating growth pattern and aggressive biological behavior. Moreover, the fact that OKC arises from the inactivation of the chromosome 9q patched gene (PTCH), a tumor suppressor gene, led in 2005 to be classified as a keratocystic odontogenic tumor. However, this denomination has never reached great acceptance, and currently, OKC has again considered a type of developing cyst instead of an unequivocal neoplasm [1]. Odontogenic cysts are epithelial-lined pathological cavities that are encircled by fibrous connective tissue and arise from odontogenic tissues that are present in the maxilla and mandible's tooth-bearing areas. Jaw cysts destroy the bone and may result in the resorption or displacement of neighboring teeth [2]. The treatment modalities are divided into conservative and radical methods. The conservative methods include strict enucleation with or without surgical scraping or cleaning utilizing a curette or marsupialization/decompression, with or without secondary therapeutic measures. The aggressive techniques include peripheral ostectomy (PO), chemical curettage with Carnoy's solution (CS), cryotherapy, electrocautery, and resection (en bloc or marginal). OKC are generally single, although they can present with multiple lesions, and are usually linked with nevoid basal cell carcinoma syndrome, which is a hereditary condition characterized by multiple basal cell skin cancers; other common signs include pits on the palms of the hands or soles of the feet, calcium deposits in the brain, developmental disability, and skeletal (bone) changes. Malignant transformation for OKC has been reported [3]. Numerous studies have documented recurrence rates of up to 62.5%, with the majority of recurrences occurring within the first 5 years following initial treatment [4]. The delicate and fragile nature of its epithelial lining, weakly connected to the capsular connective tissue, makes these lesions prone to tearing, separating, and breaking during surgical excision, sparing some epithelial remains, which can lead to recurrence since they have high proliferative activity. Recurrence may also be due to the persistence of satellite cysts that would remain during surgery, the presence of cystic debris in the adjacent bone or mucosa, or the existence of epithelial islands in the lining of mucosa [1].

## 2. Material and Methods

This descriptive prospective cross-sectional study was carried out at Khartoum Teaching Dental Hospital. Sampling technique using total coverage of the selected patients from (2012 to 2022). The following cases excluded patients who presented with other cysts, patients who refused to participate, and patients with incomplete medical records.

Method description and material used were sourcing numerical data from surgical reports, counting occurrences, and follow-up the patients for assessment of the recurrence, malignant transformation, and death.

Ethical approval was obtained from the University of Khartoum Faculty of Dentistry, Ethical Committee Review Board. Approval was obtained from the research unit in Khartoum Teaching Dental Hospital.

Data were entered in a computer master sheet using Statistical Package for the Social Sciences (SPSS) version 26. Statistical analysis was set at a 95% confidence level, 0.2, the width of the confidence interval, and the level of significance alpha is 0.05. The *χ*^2^ test was used to test the difference between categorical variables, and the Fisher's exact test was used as an alternative test in case of sparse data. Data were presented in graphs.

## 3. Results

**Figure 1:**
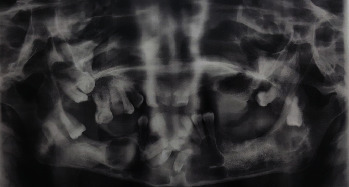
A panorama of multiple OKCs in patients of Gorlin–Goltz syndrome.

Out of 55 cases studied, the mean age at the time of diagnosis was 30 ± 17.6 years, with a slight male predilection of the disease, as 28 (50.9%) were males and 27 (49.1%) were females. About one-third (36.4%) of the 55 patients had consanguineous parents; however, only 6 (10.9%) had similar cases in the family and were diagnosed with Gorlin Goltz syndrome (Figure 1). The mean number of siblings is 2.5 ± 2.7. The disease started more than 1 year before the start of treatment in 30 (54.5%) of the patients; in other 18 (32.7%), it started less than 6 months before the start of treatment, while in the remaining 12.7%, it started 6 months to 1 year before the start of the treatment.

**Figure 2:**
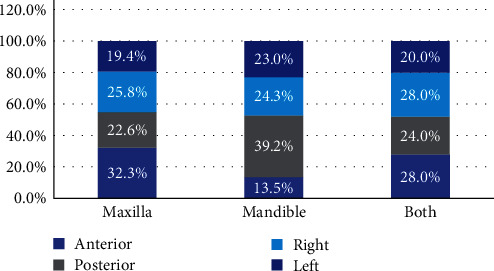
Percentage distribution of the lesion site across locations within the jaws.

Most OKCs (65%) were located within the mandible, (21.8%) were located in the maxilla, and (12.7%) were located in both the mandible and maxilla (Figure 2).

**Figure 3:**
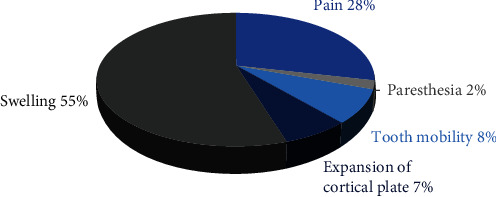
Distribution of clinical manifestation of odontogenic keratocyst across study participants.

The most common clinical manifestation was bony swelling (55%), followed by pain (28.0%), tooth mobility (7.7%), expansion of the cortical plate (6.6%), and Paresthesia (2.2%) (Figure 3).

**Figure 4:**
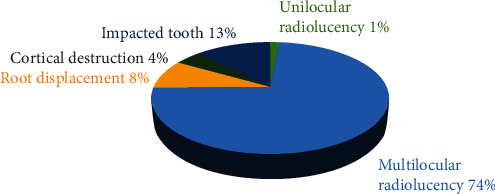
Distribution of radiological findings across study participants.

Multilocular radiolucency was the most prevalent radiological finding (73.6%), followed by impacted teeth (12.5%), root displacement (8.3%), cortical destruction (4.2%), and unilocular radiolucency (1.4%) (Figure 4).

**Figure 5:**
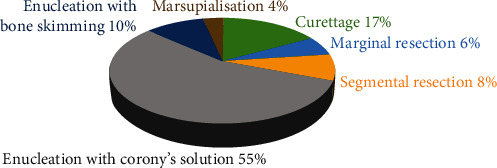
Distribution of surgical procedures.

Enucleation with CS constituted (55.2%) of all surgical procedures, followed by curettage (17.2%), enucleation with bone skimming (10.3%), segmental resection (8%), marginal resection (5.7%), and marsupialization (4%) (Figure 5).

**Figure 6:**
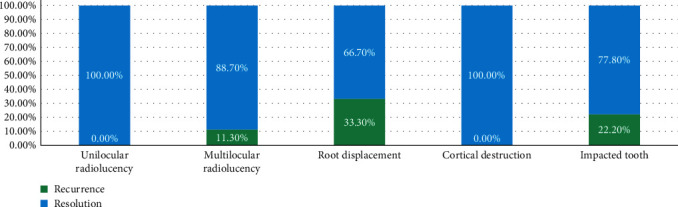
Percentage distribution of radiological findings and their relation to the recurrence.

**Figure 7:**
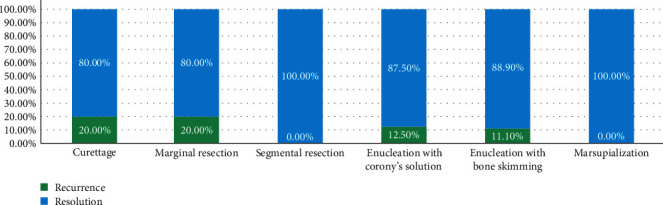
Recurrence rate of different types of surgical procedures.

Most of the patients (89.1%) were cured; only six patients (9.9%) had a recurrence. The mean duration of recurrence was 3.1 ± 2.4 years. Based on radiological findings, patients with root displacement had a 33.3% recurrence rate, followed by patients with impacted teeth (22.2%) and patients with multilocular radiolucency (11.3%) (Figure 6). Marsupialization and segmental resection had a 0% recurrence rate, while marginal resection and curettage had the highest recurrence rate of 20% (Figure 7). Patients with consanguineous parents had a higher recurrence rate (15%) compared with patients who had nonconsanguineous parents (8.6%). One out of the seven patients (14.3%) who had both mandibular and maxillary OKC had a recurrence compared with 5 (13.9%) out of the 36 patients who had mandibular OKC. None of the patients died or had a malignant transformation.

## 4. Discussion

OKC is a benign cystic lesion of the tooth; it is usually asymptomatic and slow-growing. The widespread consensus is that these lesions, like the primordial cyst, develop from leftover dental lamina [1].

The age at diagnosis and sex distributions of patients with OKC was (17–39) years, as in other studies shown by Myoung et al. [5]. Most OKCs reported they can appear at any age but more frequently in the third decade of life, as reported by Rojas et al. [6]. In the present study, OKC was found to occur in patients of a wide age range, with an average patient age of 30 ± 17.6 years. By comparing the results, the most common age is the third decade.

In the study by Kammer et al. [7], the results showed that the majority of patients were male (56.73%). Most OKCs report show a preference for males at a 1 : 1.4 ratio, as shown in the study by Tarakaji et al. [8]. In comparison our study, a total of 55 patients participated in this study of which 28 (50.9%) were males and 27 (49.1%) were females. All studies agreed on a predisposition to the disease in males. The slight difference is due to the difference in sample size.

Regarding the location of the lesion, Myoung et al. [5] reported that 60 (23.5%) and 169 cases (76.5%) out of 256 cases were discovered in the maxilla and mandible, respectively. In comparison to our study, most OKCs (65%) were located within the mandible, 21.8% were located in the maxilla and 12.7% were located in both the mandible and maxilla. Both studies agreed that the mandible is the most common location.

Clinical appearance varies greatly and is frequently characterized by soft tissue swelling without any discomfort, lesions can cause tooth displacement, aggressive growth through bone resorption, facial deformities, and spread into nearby structures [9]. In the literature review of a previous study of 256 patients by Myoung et al. [5], 118 (46.1%) experienced swelling at the time of the initial admission, 51 (19.9%) reported pain, and 42 (16.4%) had both swelling and discomfort. Borghesi et al. [10] reported that there were 17 patients (6.6%) with purulent discharge, 12 patients (4.7%) with discomfort, and 2 patients (2.1%) with paresthesia (0.8%). Fourteen individuals (5.5%) had no symptoms [11]. The present study reported that the most common clinical manifestations were bony swelling (54.9%), followed by pain (28.6%), tooth mobility (7.7%), expansion of the cortical plate (6.6%), and paresthesia (2.2%). Both studies showed that the most common clinical manifestations were bony swelling followed by pain. So similar to the previously mentioned literature, our study also had a different finding from the known fact that the OKC grows within the medullary spaces of the involved jaw without evidence of any bony swelling.

The cyst typically appears as a multilocular or unilocular radiolucency and is discovered incidentally during a routine orthopantomogram [9]. Unilocular lesions are predominant, whereas the multilocular variant is observed in approximately 30% of cases, most commonly in the mandible, as reported by Borghesi et al. [10]. However, this study is in disagreement with our study, which reported that multilocular radiolucency was the most prevalent radiological finding (73.6%), followed by impacted teeth (12.5%), root displacement (8.3%), cortical destruction (4.2%), and unilocular radiolucency (1.4%).

Various surgical options have been considered, including enucleation alone or associated with the adjunctive measures (ostectomy, CS, cryotherapy), marsupialization and decompression, marginal or segmental resection [12]. The effects of various adjunctive therapy on the peripheral lining and their ability to chemically cauterize it have been studied. After being applied for 3 min to reduce side effects, CS, which contains 1 g of ferric chloride (FeCl3) mixed in 6 ml of alcohol, 3 ml of chloroform, and 1 ml of glacial acetic acid, has been used extensively as an adjuvant treatment with a recurrence rate of about 11%. However, the US Food and Drug Administration has forbidden its use since 2013 after outlawing chloroform, which was found to be carcinogenic. Therefore, modified CS (MCS) was developed with a structure similar to CS without chloroform. Recently, 5-fluorouracil (5FU) was made available for topical usage due to its antimetabolic impact, which results in cell apoptosis [9].

Most KCOTs were treated with surgical enucleation, which is the traditional technique (to remove the lesion whole from within the bone) with CS, as shown by Dias et al. [11]. This result ties well with our study; most KCOTs were treated with surgical enucleation with CS, constituted 55.2% of all surgical procedures, followed by curettage (17.2%), enucleation with bone skimming (10.3%), segmental resection (8%), marginal resection (5.7%), and marsupialization (3%).

The KCOTs' propensity to reappear after treatment is one of their clinical traits that make therapy difficult. There are numerous logical reasons why OKC recurs continually and needs detailed surgical planning and performance [5]. Various studies have reported recurrence rates of up to 62.5% [4].

Seventy-seven of the 132 patients who were followed up in the study conducted by Myoung et al. [5] developed recurrences. The recurrence rate was 58.3%, and nine of these patients (11.7%) experienced two or more recurrences. The reported recurrent rate of OKCs by Borghesi et al. [10] after surgery is wide, up to 30%, with most recurrences occurring after conservative treatments of simple lesion enucleation [6].

In a systematic review of the literature, Johnson et al. [13] showed that enucleation is associated with the highest recurrence rate of about 30%, followed by marsupialization alone (approximately 18% recurrence rate). The association of lesion's enucleation with adjunctive technique of chemical cauterization with CS, a mixture of chloroform, absolute ethanol, glacial acetic acid, and ferric chloride, significantly reduced the recurrence rates to about 8% [13]. Surgical resection, both marginal and segmental, is related to the lowest recurrence rate but, because of its morbidity, is not recommended as a primary treatment modality and should be reserved for retreatment of patients suffering from multiple recurring lesions [13].

A previous study of the disease showed by Dias et al. [11] that recurrence rates have been estimated to be between 3% and 60%.

The present study showed that 6 patients (10.9%) out of 55 patients had a recurrence of the disease; these results are compatible with the previous studies of the disease in which recurrence rates have been estimated to be up to 62.5% [4]. The risk of recurrence may be higher with OKC of syndromic behavior, with extensive root involvement in the lesion and with soft tissue involvement, particularly in cases of cortical destruction, as the periosteum with its connective tissue origin may be involved by the epithelium of the OKCs and increases the risk of recurrence. So, the wide excision of the nearby periosteum and soft tissues may be warranted for OKCs with cortical involvement [14]. Regarding the clinical observation of root proximity to the lesion, Cunha et al. [15] reported that OKCs with root involvement had a higher rate of recurrence and assumed that the cystic line of the capsule may intermingle between the roots, causing their recurrence. In regards, the apical third root resection might be neglected with a careful preoperative assessment of OKCs to diminish relapse of them due to the involvement of the roots by the cystic epithelium, and so tooth extraction may be recommended in the particular lesions [16]. Notably, Noy et al. [17] studied the recurrence incidence of syndromic OKCs compared with non-syndromic OKCs and reported that there was an increased risk of getting recurrence in nevoid basal cell carcinoma syndrome whatever the type of surgical treatment carried out. Moreover, the literature showed that the addition of the CS to the cyst cavity for 3–5 min after surgical enucleation results in a recurrence rate lower compared to that of resection without necessarily aggressive surgery during the 3 years follow-up period as most of the recurrent OKCs are diagnosed before the third postoperative year [18, 19].

Although OKC are benign lesions, carcinomatous transformation has been described with an incidence that ranges from 0.13% to 3%, as reported by Borrás-Ferreres et al. [3]. On the contrary, our study did not show any case of malignant transformation or death. Gardner proposed specific criteria for the diagnosis of squamous cell carcinoma (SCC) arising within the odontogenic cysts: a histological transition area from benign cystic epithelial lining to frank SCC, no malignant changes in the overlying epithelium and no source of SCC in the nearby structures [20, 21]. In clinical practice means, there are no clinical carcinomatous changes on the mucosa surrounding OKC, nor was there any other malignant primary tumor.

After enucleation and PO, histopathologically confirmed OKC was given 5FU, CS, or MCS as supplementary therapy. Safety (measured as adverse events) and efficacy (reported as recurrence) were the outcomes of interest. The findings demonstrate that only patients receiving MCS experienced recurrence. The majority of reported adverse effects were paraesthesia, which might either be permanent (in the CS and MCS therapy groups) or temporary (across all supplementary therapies). Both MCS and 5FU are promising substitute adjunctive therapy now that CS is illegal. We believe that 5FU, which had the fewest adverse effects and the lowest recurrence rate, is the best option from a safety and effectiveness standpoint [9].

The three most effective treatments in reducing the recurrence rate were enucleation (E) + PO + 5FU (98.1%; very low-quality evidence), resection (83.5%; very low-quality evidence), and enucleation (E) ± PO +CS (63.8%; moderate quality evidence). Until proven otherwise, the results of this study indicate that CS continues to be the most effective fixative agent after enucleation and PO. Additionally, 5FU seems to be a successful technique with encouraging outcomes that need more study. Finally, the effectiveness of MCS is still debatable; further in vivo and in vitro investigations are necessary to develop new protocols [22].

Patients with consanguineous parents had a higher recurrence rate may due to: first, as the disease runs in families, there is more close follow-up for the members of the same family. The close follow-up makes recurrences more likely to be reported. Second, most cases of the disease with consanguinity and a high recurrence rate have parakeratinized epithelium. Third, the disease may be an incomplete form of syndrome. The association of consanguinity to disease recurrence is an area of study.

## 5. Recommendations

Future target therapy and gene therapy research may create novel therapeutic approaches to lessen the occurrence, expansion, and recurrence of OKC. The maxillofacial surgeon should be aware that until that time, small-sized OKC is asymptomatic, making it so that most patients only become aware of the lesion when swelling, facial distortion, and infection-related pain start to emerge as a result of increased lesion size. Additionally, as the size of the lesion grows, the patient's tissue deficit grows as well, which raises the rate of recurrence. A periodic radiography check is therefore required to detect the lesion before symptoms appear. Only a few studies have been done to prevent recurrence, although continuous follow-up is crucial to monitor the recurrence of OKC. There is a need for new therapeutic approaches that can reduce recurrence, like gene therapy for the PTCH gene, molecular studies (biomarkers; MCM3 and Ki67), and smooth end receptors.

Future studies should look more closely at syndromic conditions like Gorlin-Goltz syndrome and latent OKC.

## 6. Conclusion

The present study concluded that there's a slight male predilection, and the mean age was found to be 30 ± 17.6 years. It was found that the lesion began a year before admission. Swelling was the most frequent clinical symptom, followed by pain. The most common location for the cyst was the mandible and more frequently in the posterior mandible. Multilocular radiolucency was the most prevalent radiological finding. The most common surgical procedure, enucleation with CS, was employed on more than half of the patients, and only six patients experienced a recurrence. None of the patients died or developed cancer.

## Data Availability

The datasets used and/or analyzed during the current study are available from the corresponding author upon reasonable request.
